# UNDERSTANDING MOBILITY, HEALTH, AND WELL-BEING OF OLDER ADULTS USING SENSING TECHNOLOGIES

**DOI:** 10.1093/geroni/igac059.488

**Published:** 2022-12-20

**Authors:** Christina Röcke, Minxia Luo, Hans-Werner Wahl

## Abstract

Mobility has been identified as one important ingredient to older adults’ health and well-being and is considered a high priority in the global agenda of healthy and active aging. However, mobility is still a relatively understudied concept in aging research. This symposium, including three empirical studies and one concept paper, presents how different sensing technologies can be utilized to examine mobility, health and well-being in older adults. Using infrared motion sensors and contact sensors, Wu and colleagues examine indoor mobility and show its associations with physical, cognitive, and mental health in community-dwelling older adults living alone. Luo and colleagues use a custom-built mobile GPS sensor and a smartphone-based ambulatory assessment to examine daily mobility and well-being in community-dwelling older adults. They find that a day with larger life space area, more time spent in passive transport modes, and higher number of different locations is associated with higher daily life satisfaction. Similarly, using a GPS sensor combined with a smartphone-based ecological momentary assessment, Kamalyan and colleagues examine life-space mobility, social interactions, and well-being in older adults with and without HIV. They show that prior day’s at-home time is negatively associated with current day’s happiness and that prior day’s social interactions diminishes this association. Jansen presents a project combining sensor-based movement data, GPS-based geolocation data, and experience sampling to investigate relations between life-space mobility and social participation and the role of cultural and climatic differences across several European countries. Hans-Werner Wahl will discuss all papers from an ecological and contextualized aging perspective.

